# The guidewire-guided endoscopic retrograde direct cholangioscopy facilitating the difficult biliary cannulation of small papilla

**DOI:** 10.1055/a-2602-3230

**Published:** 2025-06-26

**Authors:** Shan-Shan Hu, Xiao-Gang Liu, Jie Hou, Wei-Hui Liu

**Affiliations:** 1 89669 Department of Gastroenterology and Hepatology, Sichuan Provincial Peopleʼs Hospital, School of Medicine, University of Electronic Science and Technology of China, Chengdu, China

Our team successfully developed a novel endoscopic retrograde direct cholangioscopy (ERDC) technique, which involves attaching a conical transparent cap to the front end of the cholangioscope, enabling intubation under direct vision 1 2 . However, in clinical practice, we observed variations in intubation success rates depending on papillary morphology 3 . For smaller, more challenging papillae, we learned from the traditional endoscopic retrograde cholangiopancreatography (ERCP) intubation method 4 , introducing a wire-guided ERDC strategy that significantly improves success rates for small papillae ( Video 1 ).

The guidewire-guided endoscopic retrograde direct cholangioscopy facilitating the difficult biliary cannulation of small papillaVideo 1

A male patient presented with an unexplained thickening wall of the common bile duct (CBD). ERDC was planned for direct visualization and comprehensive examination of the CBD. During duodenoscopy, the papillary orifice was found too small, preventing the conical transparent cap at the distal end of the choledochoscope from entering the papilla, resulting in failed ERDC intubation. To overcome this, we extended the guidewire tip approximately 2 mm beyond the conical transparent cap ( Fig. 1 ), inserted it into the papillary opening along the CBD’s direction, and then slipped into the CBD ( Fig. 2 ). With the guidance of guidewire, the choledochoscope able to be inserted into the small papilla ( Fig. 3 ). When the guidewire was inserted forward, the yellow bile was observed under direct vision, thus cholangioscope could be cannulated into the CBD ( Fig. 4 ). This approach successfully enabled full visualization and biopsy sampling of the distalCBD ( Fig. 5 ).

**Fig. 1:**
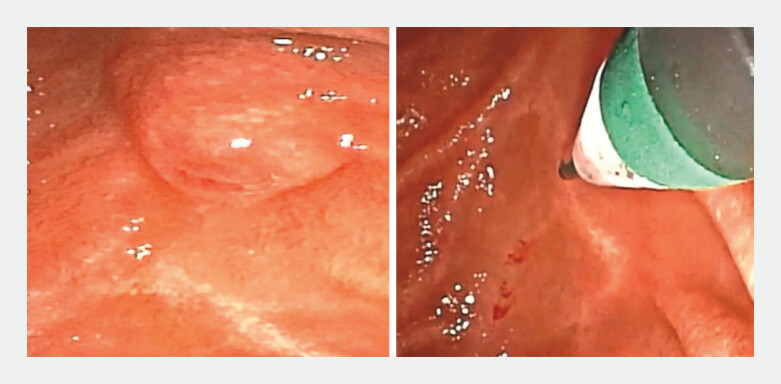
A conical transparent cap was attached to the distal end of the choledochoscope, with the guidewire tip extending approximately 2 mm beyond the cap.

**Fig. 2:**
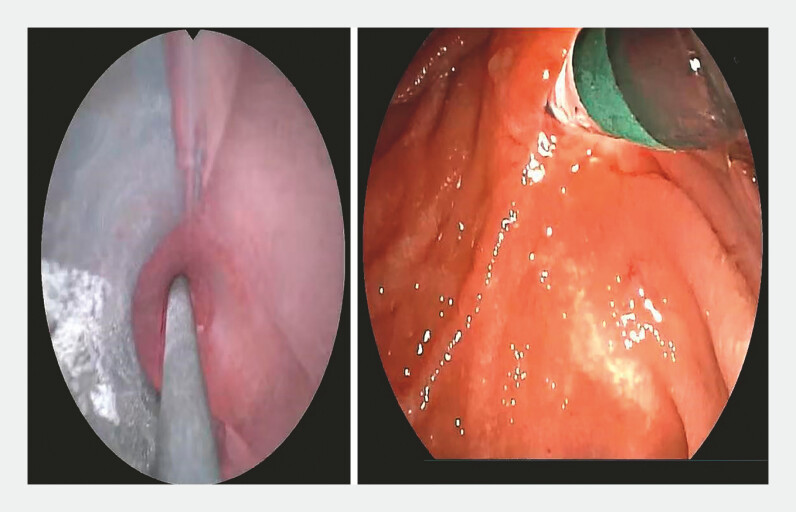
The guidewire was first inserted into the papillary orifice.

**Fig. 3:**
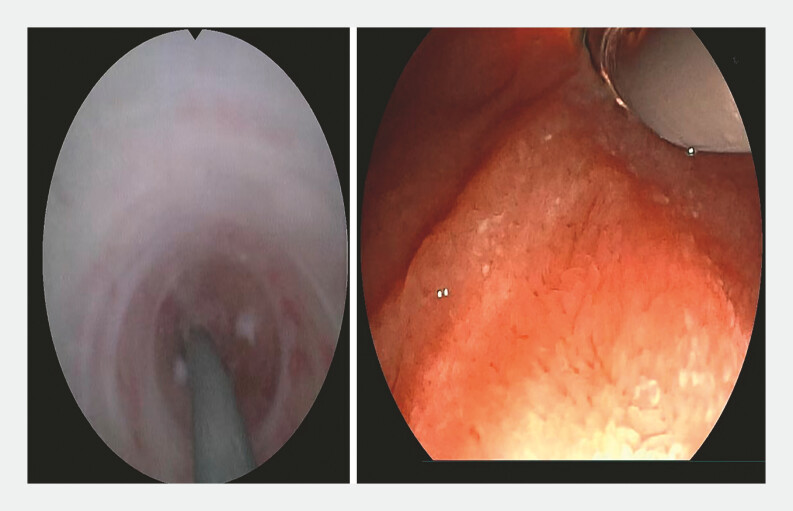
The guidewire was advanced into the common bile duct.

**Fig. 4:**
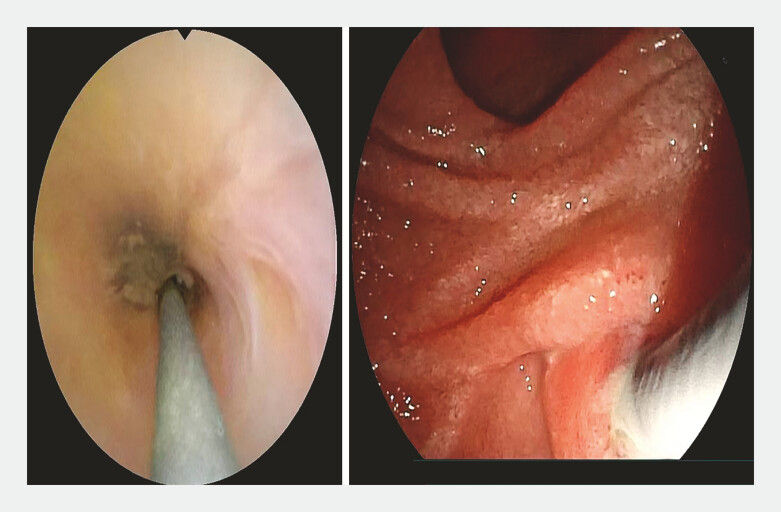
The guidewire was manipulated back and forth, allowing bile to flow out, confirming entry into the bile duct.

**Fig. 5:**
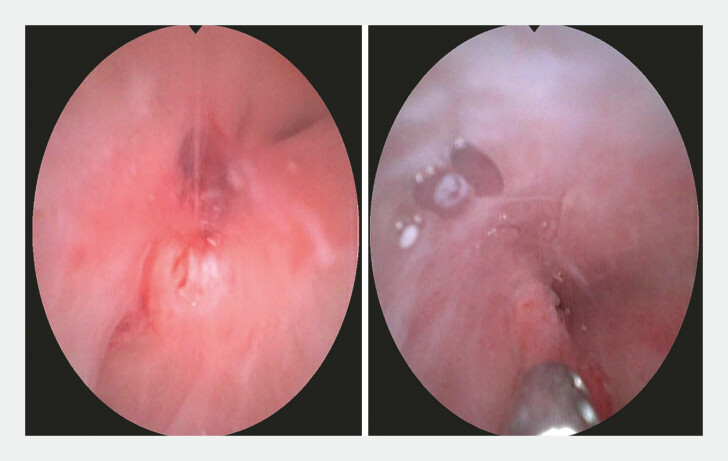
The choledochoscope was smoothly advanced along the guidewire into the common bile duct, enabling full visualization and biopsy at the lesion site.

For cases with a small papilla or when the choledochoscope's distal end exceeds the papillary orifice, this method is particularly useful. The guidewire plays a crucial guiding role, potentially improving the success rate of difficult ERDC cannulation in the future.

Endoscopy_UCTN_Code_TTT_1AR_2AB

## Notes

Endoscopy E-Videos https://eref.thieme.de/e-videos
*E-Videos* is an open access online section of the journal *Endoscopy* , reporting on interesting cases and new techniques in gastroenterological endoscopy. All papers include a high-quality video and are published with a Creative Commons CC-BY license. Endoscopy E-Videos qualify for HINARI discounts and waivers and eligibility is automatically checked during the submission process. We grant 100% waivers to articles whose corresponding authors are based in Group A countries and 50% waivers to those who are based in Group B countries as classified by Research4Life (see: https://www.research4life.org/access/eligibility/ ). This section has its own submission website at https://mc.manuscriptcentral.com/e-videos .
